# The Effects of Housework on the Health of Retired Older Adults: A Preliminary Investigation from the Tongji-Dongfeng Cohort Study, China

**DOI:** 10.1371/journal.pone.0057232

**Published:** 2013-03-01

**Authors:** Xiaopiao Wen, Yuan Liang, Jiang Zhu, Tangchun Wu

**Affiliations:** 1 Department of Social Medicine and Health Management, School of Public Health, Tongji Medical College, Huazhong University of Science and Technology, Wuhan, China; 2 Department of Health Care, Dongfeng Central Hospital, Dongfeng Motor Corporation and Hubei University of Medicine, Shiyan, China; 3 MOE Key Lab of Environment and Health, School of Public Health, Tongji Medical College, Huazhong University of Science and Technology, Wuhan, China; Oregon Health & Science University, United States of America

## Abstract

**Background:**

The aim of the current study was to explore the relationship between housework and health of retired older adults, and to provide new evidences and clues for the effects of housework on health.

**Methodology/Principal Findings:**

The data came from the baseline survey of the Tongji-Dongfeng Cohort Study with 25,501 participants. The independent variable was housework including child care, elder care, grocery shopping & cooking, and washing clothes & cleaning house. The dependent variable was self-reported two-week illness. Age, education, marital status, smoking and drinking were used as potential confounding variables. There are gender differences in the four types of housework, with higher prevalence among women than among men. The prevalence of two-week illness in women was higher than that in men. After adjusting for potential confounding variables, the four types of housework had almost no significant effects on two-week illness, except for washing clothes & cleaning house with a negative effect for women (OR = 1.17, 95% CI 1.06–1.29).

**Conclusions/Significance:**

The association of housework with health, especially of child care, elder care, and grocery shopping & cooking without significance and of washing clothes & cleaning house with significance for women, would provide a valuable clue for in-depth study of housework, namely the effects of housework on health would be subdivided into the dimensions of psychological and social activity besides physical activity, and it may not be suitable to simply classify housework as a kind of exercise.

## Introduction

Population aging, longer life expectancy, and relatively earlier retirement ages have enhanced interest in research on lifestyle and health for older adults [1]–[3]. Compared with adults in their prime, the “workplace” of retired older adults has undergone major changes as their places of residence have become the location of their major activities and, accordingly, housework has become their main work. Much recent research has explored the division of housework between husbands and wives, especially with young married couples [4]–[9], while few studies have focused on older adults [1], [10]–[11].

In recent decades, most of the studies on housework have focused on gender inequality in young married couples and prime-age adults. Several sociological theories have been developed as explanations of gender inequality, including the relative resource model, the gender ideology model, the time availability model and so on [7]–[9], [12]. Although the division of housework in the United States and European countries has become more equal over time (partly because men’s housework time has increased, but more because women’s involvement in the labor market has greatly increased and their housework time has decreased), men’s contribution to housework remains lower than women’s [13]–[16].

Some studies suggest a positive association between perceived unfairness in the division of housework and depressive symptoms for women [7], [17]–[18]. In addition, the results of the Barcelona Health Interview Survey (2000) suggest that hours of housework per week were associated with poor self-reported health status among women [19]. Although housework could be burdensome, it has some positive aspects [20]–[22]. Compared to employment, housework offers more autonomy. Moreover, housework could be productive, involves physical activity, and yields a clean and pleasing living environment, all of which could reduce psychological distress. Faced with these inconsistent and conflicting results, in-depth studies are necessary to demonstrate an independent health benefit of involvement in housework and should include international and cross-cultural comparisons [5]–[9], [16].

In research on the health of older adults, most of the studies have focused on socio-economic status, utilization of health services and health care, and health-risk behaviors, including smoking, alcohol consumption and physical activity. A solid evidence base supports the positive relationship between regular physical activity and health, and studies have largely examined the effects of brisk walking, leisure time exercise, or occupational activity rather than domestic activities [21], [23]–[26]. There is very little evidence about housework and the health of older adults [11]. To our knowledge, only one study from the United Kingdom was about the effects of housework on the health of older adults. Results from the British Women’s Heart and Health Study with 2341 women aged 60 to 79 showed that heavy housework was not associated with reduced levels of being overweight [11].

In China, due to traditional Confucian ideology [7], women are the main bearers of housework both in urban and in rural areas. At present, there are only a few studies on housework, mainly conducted with prime-age adults and few with older adults [27]–[29]. In addition, the time periods of retirement in China, except for early retirement due to illness and injury, are in general 60–65 years old for males and 50–55 years old for females, so the majority of the retired are older adults. For the retired older adults, family is their main arena and the housework becomes their main work. Since China is a developing country with the largest population and a rapidly aging population, studies of retired older adults in China would be of value not only to the Chinese, but also to the people in other developing countries.

To address the above issues, the current study offers empirical evidence about housework and retired older adults in China. The aim of the current study was to explore the relationship between housework and health of retired older adults, and to provide new evidences and clues for the effects of housework on health.

## Methods

### Participants

The data came from the baseline survey of Tongji-Dongfeng Cohort Study [30]. The aim of the cohort study was to examine a wide range of factors behind the causes and progression of chronic diseases and the potential role of gene–environment interactions, and the current study is part of the whole cohort study. A total of 31,000 retired employees were invited at the Dongfeng Motor Corporation, DMC. For those who did not respond to the invitation, the staff at the Retirement Office followed them up with a telephone call. Approximately 87% (*n = *27,009) of the invited participants agreed and provided baseline blood samples and questionnaire information between September 2008 and June 2010. Among those who did not participate in this study, most moved to other cities. The Medical Ethics Committee of the School of Public Health, Tongji Medical College, Huazhong University of Science and Technology, HUST, and Dongfeng General Hospital, DMC, has approved this study. All participants provided written informed consent. The effective questionnaire of the current study was 25,501, and the valid rate was 94.42% (25,501/27,009).

### Measures

#### Independent variable

The independent variable was housework in the last six months. Most researchers agree that major housework includes cooking, cleaning, shopping for groceries and household goods, doing dishes, and laundry. In addition, comparing China’s traditional family with families in modern Western countries, there was this large difference: many Chinese older adults lived together with their children (including married adult children), and even their parents, which was due to both socio-economic status and the Chinese culture of filial piety of which the latter may be the more important. Combined with previous studies on housework in China [7], [27]–[29], four types of housework were included in the current study: namely, child care, elder care, grocery shopping & cooking, and washing clothes & cleaning house. Housework was surveyed by asking: “In the last six months, have you often done the following household chores?” Yes or No was used for each type of housework.

#### Dependent variable and control variables

The dependent variable was self-reported two-week illness, which was continuously used in the China National Health Services Survey from the first (1993) to the fourth (2008) with a cycle of every five years [31]. Self-reported two-week illness was surveyed by asking: “Have you had any physical and mental discomforts during the last two weeks?” The socio-demographic variables, including age, education, marital status, and health-related behavior, including smoking and drinking, were used as the control variables.

### Statistical Analysis

Data was analyzed using the SAS statistics package, release 8.2 (SAS Institute Inc., Cary NC). We constructed logistic regression models to analysis the effects of housework on prevalence of two-week illness. Unadjusted and adjusted OR and 95% CI were calculated using univariable and multivariable regression analysis, respectively. Two logistic regression models were conducted separately for male and female. Two-sided tests were used for all analyses, and p-values of 0.05 or less were considered statistically significant.

## Results

The socio-demographic characteristics of the 25,501 subjects are shown in Table 1. The sample comprised 11,379 (44.62%) men aged 33 to 104 years (Mean ± SD: 66.27±6.71) and 14,122 (55.38%) women aged 34 to 104 years (Mean ± SD: 61.36±7.91). The reason why the number of female is more than that of male is mainly due to the cohort sample, namely the retired employees of the DMC, and the periods of retirement in China are in general 60–65 years old for males and 50–55 years old for females. In other words, women retire earlier than men. Student’s T test showed that the men were older than the women (T = 53.62, p<0.0001). Gender differences were also found with religion, education and married status. Table 2 shows that women had a higher prevalence of child care, elder care, grocery shopping & cooking, and washing clothes & cleaning house than men (29.79% vs. 25.99%; 43.23% vs. 19%; 88.55% vs. 62.95%; 81.74% vs. 46.15%; respectively). In addition, women had a higher prevalence of two-week illness than male (52.02% vs. 49.96%). Table 3 presents unadjusted and adjusted OR and 95% CI for the associations between independent variables and two-week illness, and the results of univariable analysis were very similar to those of multivariable analysis. After adjusting for potential confounding variables, the four kinds of housework had almost no significant association with two-week illness, except for doing laundry and cleaning house in women, which had a negative association (OR = 1.17, 95% CI: 1.06–1.29).

**Table 1:** Descriptive Statistics of Socio-demographic variables (n = 25,501).

Socio-demographic variables	Total		Male		Female		*X* ^2^	*P*
	N	%	N	%	N	%		
**Gender**								
male	11379	44.62						
female	14122	55.38						
**Age**								
33–59	7498	29.40	1022	8.98	6476	45.86	4165.7313	<.0001
60–64	7391	28.98	4154	36.51	3237	22.92		
65–69	4952	19.42	3013	26.48	1939	13.73		
70–74	3366	13.20	1848	16.24	1518	10.75		
75–79	1797	7.05	1050	9.23	747	5.29		
80–104	497	1.95	292	2.57	205	1.45		
**Education**								
Primary and below	7534	29.54	3260	28.65	4274	30.26	470.3388	<.0001
Junior school	9154	35.90	3933	34.56	5221	36.97		
High School	6136	24.06	2472	21.72	3664	25.95		
College and above	2677	10.50	1714	15.06	963	6.82		
**Ethnicity**								
Han nationality	25126	98.53	11207	98.49	13919	98.56	0.2387	0.6252
Others	375	1.47	172	1.51	203	1.44		
**Religion**								
Yes	352	1.38	114	1.00	238	1.69	21.6237	<.0001
No	25149	98.62	11265	99.00	13884	98.31		
**Marital status**								
Divorced/Never married	560	2.20	175	1.54	385	2.73	538.6841	<.0001
Married	22307	87.48	10361	91.05	11946	84.59		
Widowed	2029	7.96	464	4.08	1565	11.08		
Remarried	605	2.37	379	3.33	226	1.60		

**Table 2:** Descriptive Statistics of Housework and two-week illness (n = 25,501).

Housework	Total		Male		Female		*X* ^2^	*P*
	N	%	N	%	N	%		
**Child care**								
Yes	7164	28.09	2957	25.99	4207	29.79	45.1377	<.0001
No	18337	71.91	8422	74.01	9915	70.21		
**Older care**								
Yes	8126	31.87	2162	19.00	5964	42.23	1566.5042	<.0001
No	17375	68.13	9217	81.00	8158	57.77		
**Grocery shopping & cooking**								
Yes	19627	76.97	7122	62.59	12505	88.55	2395.5532	<.0001
No	5874	23.03	4257	37.41	1617	11.45		
**Washing clothes & cleaning house**								
Yes	16794	65.86	5251	46.15	11543	81.74	3549.9532	<.0001
No	8707	34.14	6128	53.85	2579	18.26		
**Two-week illness**								
Yes	13015	51.10	5677	49.96	7338	52.02	10.6420	0.0011
No	12453	48.90	5685	50.04	6768	47.98		

**Table 3:** Unadjusted and adjusted associations between housework and two-week illness in retired older adults (n = 25,501).

Variables	Male			Female	
	Unadjusted OR(95%CI)	Adjusted OR(95%CI)		Unadjusted OR(95%CI)	Adjusted OR(95%CI)
**Child care**					
Yes	1.02 (0.94–1.11)	1.09 (0.99–1.19)		1.02 (0.94–1.09)	1.04 (0.96–1.12)
No	1.00	1.00		1.00	1.00
**Older care**					
Yes	0.95 (0.87–1.04)	0.95 (0.86–1.05)		1.02 (0.95–1.09)	1.04 (0.97–1.12)
No	1.00	1.00		1.00	1.00
**Grocery shopping & cooking**					
Yes	1.02 (0.95–1.10)		0.98 (0.90–1.07)	0.95 (0.86–1.06)	0.90 (0.80–1.01)
No	1.00		1.00	1.00	1.00
**Washing clothes & cleaning house**					
Yes	1.05 (0.98–1.13)		1.03 (0.95–1.12)	1.10 (1.01–1.20)*	1.17 (1.06–1.29)**
No	1.00		1.00	1.00	1.00
**Age**					
80–104	1.20 (0.93–1.56)		1.15 (0.88–1.50)	1.82 (1.37–2.42)***	1.96 (1.46–2.63)***
75–79	1.45 (1.22–1.72)***		1.34 (1.12–1.61)**	1.56 (1.34–1.81)***	1.64 (1.39–1.94)***
70–74	1.39 (1.19–1.62)***		1.26 (1.08–1.48)**	1.62 (1.45–1.81)***	1.70 (1.51–1.92)***
65–69	1.25 (1.09–1.44)**		1.17 (1.01–1.35)*	1.64 (1.48–1.81)***	1.70 (1.53–1.89)***
60–64	1.09 (0.95–1.25)		1.04 (0.90–1.19)	1.36 (1.25–1.48)***	1.40 (1.28–1.53)***
33–59	1.00		1.00	1.00	1.00
**Education**					
College and above	1.37 (1.21–1.54)***		1.38 (1.23–1.56)***	1.21 (1.05–1.39)**	1.35 (1.17–1.59)***
High School	1.27 (1.14–1.41)***		1.29 (1.16–1.43)***	0.95 (0.87–1.04)	1.12 (1.02–1.23)*
Junior school	1.05 (0.96–1.15)		1.08 (0.98–1.18)	0.88 (0.81–0.96)**	1.00 (0.92–1.09)
Primary and below	1.00		1.00	1.00	1.00
**Marital status**					
Divorced/Never married	0.78 (0.58–1.06)		0.84 (0.62–1.15)	0.99 (0.81–1.22)	1.10 (0.90–1.36)
Widowed	1.13 (0.94–1.36)		1.08 (0.89–1.31)	1.18 (1.06–1.31)**	1.04 (0.93–1.17)
Remarried	1.19 (0.97–1.46)		1.26 (1.02–1.55)*	0.97 (0.75–1.27)	1.04 (0.79–1.35)
Married	1.00		1.00	1.00	1.00
**Smoking**					
Former smoker	1.54 (1.4–1.69)***		1.41 (1.27–1.56)***	1.64 (1.15–2.35)**	1.31 (0.91–1.89)
Current smoker	1.00 (0.92–1.09)		1.05 (0.95–1.15)	1.19 (0.96–1.49)	1.02 (0.81–1.27)
Never smoker	1.00		1.00	1.00	1.00
**Drinking**					
Former drinker	1.90 (1.68–2.15)***		1.74 (1.52–1.98)***	1.92 (1.37–2.70)***	2.05 (1.46–2.90)***
Current drinker	0.97 (0.90–1.05)		0.99 (0.91–1.08)	1.29 (1.13–1.49)***	1.41 (1.22–1.62)***
Never drinker	1.00		1.00	1.00	1.00

Note: *P<0.05;

** *P<*0.01;

*** *P<*0.001 (two-tailed test).

In addition, age and education level had significant associations with two-week illness, whether in men or in women. For men, former smokers and former drinkers are more likely to report two-week illness in comparison with never smokers and never drinkers (OR = 1.41, 95% CI: 1.27–1.56, p<0.001; OR = 1.74, 95% CI: 1.52–1.98, p<0.001, respectively); for women, both current and former drinkers were more likely to report two-week illness (OR = 1.41, 95% CI: 1.22–1.62, p<0.001; OR = 2.05, 95% CI: 1.46–2.90, p<0.001, respectively), but there was no association with smoking.

## Discussion

To our knowledge, the current study provided the first empirical data about housework and its associations with self-reported two-week illness in retired older adults in China. The results showed that there are gender differences in the four types of housework, with higher prevalence among women than among men. The prevalence of two-week illness in women was higher than that in men. However, the association of the four types of housework with self-reported health was almost not significant, except for washing clothes & cleaning house with a negative effect for women.

As to gender differences in housework, the results of the current study confirmed those of the previous studies [5]–[7], [12], [15], [16]. However, there is a problem worth noting. Although there are some theories about gender differences of housework, such as the relative resource model, the time availability model, and the gender ideology model and so on, they are mainly aimed at prime-age adults. Due to the differences in the working state of the retired, some theories might lose their premise in explaining gender differences of housework among retired older adults. Perhaps, gender differences in housework are not simply due to relative resource and time availability, which should be reflected by the results of the current study, if true. Historical, cultural and social values factors may be involved as well [7]–[8], [13].

Regarding the effects of housework on health, there are few studies at present, and the existing studies mainly targeted at prime-age adult women [18], [20]. There is very little evidence on the effects of housework among older adults [11]. According to a national survey among elderly British women [11], heavy housework was not associated with reduced levels of being overweight. The current study suggested that the association of housework with two-week illness was almost not significant. Although there are some differences with measurement tools of housework and health outcome, both results were similar to each other, namely, that the association of housework with the health of older adults was not significant in general.

In addition and perhaps more importantly, the association of housework with health in the current study, especially of child care, elder care, and grocery shopping & cooking (which were not significant) and of washing clothes & cleaning house (which were significant for women), would provide a valuable clue for in-depth study of housework. In fact, it is not difficult to perceive that the three types of housework without significant association have the common feature of interpersonal interactions; on the contrary, washing clothes & cleaning house involve almost no communication with others, and to some extent are boring. Perhaps the physical activity in housework would benefit health, but the interpersonal/social and psychological activity (co-existence of conflict and support [6], [20]) in housework would confound the association of physical activity with health. On the other hand, there may be active or passive attitudes about perceived responsibility for housework, which would also confound the association of housework with health. In other word, the features of housework perhaps would not only include the dimension of physical activity, but also the dimensions of psychological and social activity. These multi-dimensional features would be helpful for analyzing the inconsistent and conflicting health effects of housework [6], [17]–[22]. Perhaps, it may not be suitable for housework to be simply classified into a kind of exercise. Unlike general exercise, a negative attitude and interpersonal conflicts with housework would be bad for health and the contrary would be beneficial to health. A population-based study in Lebanon showed that there was a significant association between husbands’ involvement in housework and their wives’ psychosocial health, which would indirectly and partly suggest the multi-dimensional features of housework [32]. To our knowledge, there are almost no in-depth analyses of the issues mentioned above.

There are a few limitations of the current study that may reduce the generalizability of our findings. First, we used self-report measures to assess housework and health status, especially housework. Some quantitative methods, such as the time and frequency of housework and metabolic equivalent (METs), were not used in the current study [33]–[34]. Second, in general, the numbers of female older adults are greater than the numbers of male, which is mainly due to the cohort study sample [30]. The participants did not come from a community-based population, but from the retired employees of the Dongfeng automobile company, which needed males to do lots of heavy physical labor. Third, like all cross-sectional studies, it is difficult to establish causal association between independent and dependant variables. Future studies are needed to clarify these important issues.

Despite these limitations, the current study provides initial evidence of *housework and its associations with health*, especially of child care, elder care, and grocery shopping & cooking (which were not significant) and of washing clothes & cleaning house (which were significant for women), which would provide a valuable clue for in-depth study of housework. The effects of housework on health would be subdivided into the dimensions of psychological and social activity besides physical activity, and it may not be suitable to simply classify housework as a kind of exercise.
